# Assessment of a rapid diagnostic test based on loop-mediated isothermal amplification (LAMP) to identify the most frequent pathogens causing hospital-acquired pneumonia

**DOI:** 10.3389/fcimb.2025.1609666

**Published:** 2025-09-08

**Authors:** Anna Sellarès-Crous, Arturo Martínez-Trejo, Natàlia Arnalda-Muñoz, Giulia Gatti, Miriam Villanueva-López, Andrea Vergara-Gómez, Francesc Marco-Reverté, Mateu Espasa-Soley, Jordi Vila-Estapé

**Affiliations:** ^1^ Department of Clinical Microbiology, Hospital Clínic, Barcelona, Spain; ^2^ Barcelona Institute for Global Health (ISGlobal), Barcelona, Spain; ^3^ School of Medicine and Health Sciences, University of Barcelona, Barcelona, Spain; ^4^ Department of Surgical and Medical Sciences (DIMEC), Alma Mater Studiorum-University of Bologna, Bologna, Italy; ^5^ Centro de Investigación Biomédica en Red (CIBER) de Enfermedades Infecciosas (CIBERINFEC), Instituto Salud Carlos III, Madrid, Spain

**Keywords:** hospital-acquired pneumonia, LAMP, rapid diagnostic test, pathogen detection, respiratory samples

## Abstract

**Introduction:**

Hospital-acquired pneumonia (HAP) is a serious infection affecting patients in the hospital setting. This study aimed to evaluate a novel multiplex detection method using loop-mediated isothermal amplification (LAMP) technology to identify six primary bacterial pathogens responsible for HAP directly from respiratory samples.

**Methods:**

A total of 119 clinical samples were analyzed by LAMP technology, including mainly bronchoalveolar lavages, endotracheal aspirates, and bronchoaspirates.

**Results and discussion:**

The results of the LAMP and traditional culture methods showed an accuracy of 93.0%. In some discordant cases between culture and LAMP, multiplex PCR (FilmArray Pneumonia Panel) showed a strong correlation with the LAMP results, confirming the potential use of this technique as a diagnostic detection tool. The clinical sensitivity of the LAMP assay was 93.3% with a specificity of 92.0%. Correlation analysis revealed a weak negative relationship between bacterial load and time to positivity (*r* = −0.177, *p* = 0.05). This study underscores the potential of LAMP as a rapid and accurate tool for the diagnosis of HAP, facilitating the turnaround time for microbiology laboratory results, which is critical for improving the outcomes of patients with HAP.

## 1 Introduction

Hospital-acquired pneumonia (HAP) involves infection of the pulmonary parenchyma, which develops in hospitalized patients within 48 h or more after admission. It is usually caused by microorganisms present in hospital settings. Ventilator-acquired pneumonia (VAP) is a significant subset of HAP, which occurs more than 48 h after endotracheal intubation in patients in an intensive care unit (ICU) (Kalil et al., 2016; Torres et al., 2017).

Healthcare-associated infections (HAIs) are a major public health burden. HAP is the second most common nosocomial infection and the most frequent ICU-acquired infection (including VAP), and it is currently the main cause of death from nosocomial infection in critically ill patients (Blot et al., 2022; Candel et al., 2023). According to the etiological agent, *Pseudomonas aeruginosa* and *Staphylococcus aureus* are the main pathogens causing HAP, followed by *Klebsiella pneumoniae* complex and *Escherichia coli* (Candel et al., 2023; Gallego-Berciano et al., 2023). The frequency of these pathogens varies between regions and countries. In ICU patients, other non-fermenting Gram-negative bacilli, such as *Stenotrophomonas maltophilia* and *Acinetobacter baumannii*, are especially relevant (Torres et al., 2017; Candel et al., 2023; SEMICYUC, 2023).

In patients with nosocomial infections, the selection of initial empiric antibiotic therapy is crucial, particularly when resistant pathogens are involved, as it can significantly affect patient outcomes. The impact of HAIs, especially VAP, on morbidity, mortality, and healthcare costs is well-documented. Delays in administering appropriate therapy have been linked to worse outcomes, extended hospital stays, increased healthcare expenses, and higher mortality rates (Rello, 2007; Kuti et al., 2008; Bonine et al., 2019; Zasowski et al., 2020).

Traditional microbiological tests, such as Gram staining and culture, are time-consuming but remain the standard techniques for the diagnosis of respiratory infections. However, new molecular tests allow reducing the time to achieve results to only hours, enabling rapid diagnosis and facilitating patient management. There are multiple panels for the syndromic diagnosis of respiratory infections, including community-acquired, viral, or upper respiratory tract infections, but fewer panels are available for more invasive infections or those caused by bacteria resulting in HAI (Ramanan et al., 2018; Candel et al., 2023). Most panels are based on real-time polymerase chain reactions (qPCR), but other technologies, such as loop-mediated isothermal amplification (LAMP), are available for the detection of the nucleic acid material of the bacteria.

LAMP was developed in 2000 by Notomi (2000) and is a simple nucleic acid amplification method, which primarily features the amplification of nucleic acids in the sample at a constant temperature. This is achieved using a multiple set of primers that amplify the target present in the sample more efficiently and specifically due to their loop-shaped configuration. Thus, LAMP is used as an alternative to PCR for its more rapid and cost-effective detection of targets in samples and has already been used with good results for diagnosing respiratory infections, among others (Anastasiou et al., 2021; Feleke et al., 2021; Gaber et al., 2022; Jang et al., 2024; Nikolova, 2024). Additionally, LAMP can detect more than one target in a single reaction, thereby facilitating the design of syndromic panels, streamlining workload, and reducing response time in the microbiology laboratory. However, when the number of bacterial targets increases (i.e., multiplexing), there is a higher risk of primer–primer interactions, competition for reagents, and non-specific amplification, which may lead to reduced specificity and lower amplification efficiency for individual targets, ultimately impacting sensitivity. This is particularly relevant in LAMP, which uses multiple primers per target (typically four to six), increasing the complexity of multiplex designs. Conversely, assays with fewer targets typically achieve higher sensitivity and specificity (Notomi, 2000; Zhang et al., 2014; Zhou et al., 2014).

Although several studies have addressed the use of LAMP to individually detect each of the main bacterial pathogens involved in HAP from respiratory specimens (*S. aureus*, *E. coli*, *P. aeruginosa*, *S. maltophilia*, *A. baumannii*, and *K. pneumoniae*) (Lin et al., 2017; Poirier et al., 2022; Ferrusca Bernal et al., 2024), very few have evaluated these pathogens collectively. Thus, the aim of this study was to evaluate a set of pathogens causing HAP using LAMP technology. The detection panel analyzed here contributes to the limited existing literature by providing a broader and more integrated analysis of the use of panels based on LAMP, which have scarcely been investigated previously.

## 2 Materials and methods

We devised a Swift protocol for the discrimination of six distinct bacteria causing HAP using LAMP technology directly in respiratory samples. The bacteria targeted were *E. coli*, *S. aureus*, *P. aeruginosa*, *K. pneumoniae*, *S. maltophilia*, and *A. baumannii*. Subsequently, we compared the performance of LAMP with traditional culture methods. Our study included different types of specimens: bronchoalveolar lavage (BAL), endotracheal aspirate (EA), bronchoaspirate (BAS), and sputum.

### 2.1 Sample collection

Positive and negative samples were collected from the Clinical Microbiology Laboratory at the Hospital Clínic in Barcelona, Spain. A large part of the samples was collected between 2022 and 2024. The types and microbiological findings of the samples are presented in **Supplementary Table S1**.

### 2.2 Routine microbiological methods

Sterile containers were utilized to collect respiratory specimens, which were then transported to the laboratory for processing within 2 h.

To assess specimen quality, Gram staining of the specimens was performed and evaluated following the Murray–Washington criteria (Murray and Washington, 1975), selecting only good quality samples (grades 4–6) for culture and LAMP analysis. All respiratory samples were Gram-stained to determine the presence of Gram-negative bacilli, Gram-positive cocci or mixed microbiota followed by quantitative culture (blood and chocolate agar). Significant bacterial growth related to respiratory pathogens was quantified and identified utilizing mass spectrometry technology (MALDI-TOF, Bruker Daltonics, Germany). The LAMP assay was further carried out.

### 2.3 Loop-mediated isothermal amplification

**Figure 1:**
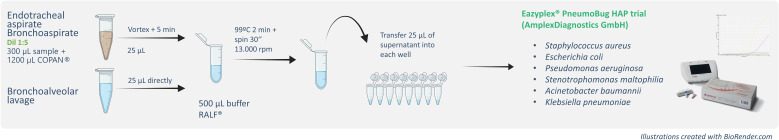
Respiratory samples for the LAMP measurement workflow. Depending on the physical characteristics of the respiratory sample, a dilution was performed if the specimen was dense, as in the case of endotracheal aspirates and bronchoaspirates. In the case of bronchoalveolar lavage, this step was omitted. The sample was then transferred to a recipient with RALF buffer and incubated for DNA extraction. After spinning, the sample was transferred to the LAMP reaction tubes, and the reaction was performed at 65°C for 25 min. Finally, the results were interpreted.

Taking into account the nature of each specimen, viscous and dense samples, including EAs and BAS, were prediluted 1:5 with Sputum Liquefying Solution (SLSolution™, COPAN, Italy) (300 µL of sample plus 1,200 µL of COPAN), a DTT-containing (dithiothreitol) liquefying solution. After gentle vortex and 5 min of incubation (or until fully liquified), 25 µL of the mixture was transferred to a recipient containing 500 µL of RALF buffer for bacterial lysis (AmplexDiagnostics, GmbH, Germany). The sample was then heated for 2 min at 99 °C for DNA extraction, followed by a brief 30-s centrifugation at 13,000 rpm. The supernatant was used for LAMP. Concerning BALs, 25 µL of the specimen was directly transferred to the RALF buffer, and then the previously described protocol was followed (**Figure 1**).

eazyplex^®^ PneumoBug test strips (AmplexDiagnostics GmbH, Germany) were used, and freeze-dried ready-to-use amplification components detected each bacterial target in individual wells. The wells were rehydrated with 25 µL of the final supernatant mentioned above. The LAMP reaction was performed in the Genie^®^ II Mk2 instrument (AmplexDiagnostics GmbH, Germany) at 65 °C for 25 min.

The following genes were used as targets for species-specific detection: *E. coli* gene *pho*A, *S. aureus* gene *fem*A, *P. aeruginosa* gene *opr*L, *K. pneumoniae* gene *pho*E, *S. maltophilia* gene *hrp*A, and *A. baumannii* gene encoding the OXA-51 β-lactamase.

### 2.4 Determination of the limits of detection

The limit of detection (LoD) was calculated for each bacterium included in the test. Bacterial suspensions were performed for each bacterial strain at different concentrations ranging from 10^6^ to 10^2^ colony-forming units (CFUs)/mL. The strains used were *S. aureus* ATCC 25923, *E. coli* ATCC 25922, *P. aeruginosa* ATCC 27853, *A. baumannii* ATCC 19606, *K. pneumoniae* ATCC 13883, and a clinical strain of *S. maltophilia*.

A 0.9% NaCl solution was used as a diluent. To determine the final concentration, the number of CFU was calculated based on the approximate concentration determined by the culture, plating 50 µL of each suspension on TSA agar and incubating at 37 °C for 18 h. After culturing the suspension, the LAMP protocol described above was followed (the use of the mucolytic was omitted), adding 25 µL of bacterial suspension to 500 µL of RALF buffer and following the next steps described for the reaction. Each suspension was analyzed in triplicate. The dilution performed with the RALF buffer was considered for the final CFU/mL determination.

### 2.5 BioFire^®^ FilmArray^®^ Pneumonia Panel protocol

To analyze discrepancies between culture and LAMP results, the BioFire^®^ FilmArray^®^ Pneumonia Panel Plus v2.0 system (BioFire^®^ Pneumonia Panel Plus 2.0, BioFire Diagnostics, bioMérieux, Marcy l’Étoile, France) was used for microbial identification. Sample processing followed the manufacturer’s instructions. The sample was inoculated into the injection vial using the manufacturer-provided swab, which contains the buffer required for proper homogenization of the sample prior to panel injection. Before sample loading, the reaction panel was pretreated with the hydration solution. Subsequently, the panel was placed into the FilmArray^®^ instrument, in which the reaction was carried out. Each sample was processed individually to maintain analytical integrity.

### 2.6 Statistical analysis

The sensitivity, specificity, positive predictive value (PPV), and negative predictive value (NPV) as described elsewhere (Altman and Bland, 1994; Park et al., 2024) and the accuracy of the LAMP panel were calculated using the MedCalc software for Windows Ver. 23.0.2 (MedCalc Software, Ostend, Belgium). Major errors were characterized as instances when the microorganism identified by LAMP differed entirely from those identified in culture, or when LAMP failed to detect a pathogen that had grown in culture. Minor errors were described as cases for which the main pathogen was correctly identified, but LAMP also detected additional microorganisms.

A correlation study was performed to determine the association between LAMP detection time and pathogen concentration in the clinical samples, as determined by quantitative culture in clinical samples. Negative samples were excluded, and only cases with the same culture and LAMP results were included. In samples in which multiple microorganisms were detected and culture results aligned with LAMP identification, the values of each microorganism were individually analyzed. Log10 CFU/mL value detection time in total minutes was used for consistency and to ease the result analysis. Data normality was evaluated using the Shapiro–Wilk test, which is adequate for small and moderate sample sizes such as those in the present study, followed by a one-tailed Spearman correlation analysis given that the data obtained did not follow a normal distribution. The *p*-value for statistical significance was <0.05. Additionally, to observe the statistical dispersion of the detection time, the median and the interquartile range (IQR) for each of the different pathogens detected by LAMP were determined. The statistical analyses were conducted using the R Studio software package (ver. 4.4.3), using the same software for the generation of graphs.

## 3 Results

### 3.1 LAMP compared to the standard protocol

A total of 119 respiratory samples were collected: 61 EAs, 43 BAS, 13 BALs, and 2 sputum samples. In 105 out of 119 cases (88.23%), LAMP results were fully concordant with those of conventional culture: in 82 samples, the result was positive in both tests, while in 23 samples, the result was also negative in both assays. Notably, in 7 positive samples, a two- or five-fold dilution with COPAN was required to validate the internal control.

Nine major errors were detected: in six samples, the LAMP result was negative, while the culture was significantly positive; in two samples, the rapid test only detected one of the two microorganisms identified in the culture, and in one sample, the culture result was negative, while molecular detection was positive. On the other hand, in four samples, an additional microorganism was detected by LAMP apart from the microorganism isolated in the culture. In one sample, culture detected *Morganella morganii*, but the LAMP kit detected *S. aureus*. These five samples were classified as minor errors (**Table 1**).

**Table 1:** Results obtained by eazyplex^®^ PneumoBug HAP test strips according to the results obtained by conventional culture.

Results of the conventional culture	N	Concordance	Minor errors	Major errors	Comments
*P. aeruginosa*	38	33	4^a^	1	^MiE^In four samples, extra microorganisms were also detected, which were *S. aureus* + *K. pneumoniae* (one sample), *E. coli + S. aureus* (one sample), *K. pneumoniae* (one sample), and *S. maltophilia* (one sample). ^MaE^In one sample, *P. aeruginosa* was not detected.
*K. pneumoniae* complex	14	12	0	2	^MaE^In two samples, *K. pneumoniae* was not detected by LAMP.
*S. aureus*	16	15	0	1	^MaE^In one sample, *S. aureus* was not detected by LAMP.
*E. coli*	8	8	0	0	
*S. maltophilia*	11	9	0	2	^MaE^In two samples, *S. maltophilia* was not detected.
*A. baumannii*	3	3	0	0	
Negative^b^	25	23	1^1^	1^1^	^MiE^In one sample, *S. aureus* was detected by LAMP, whereas culture was only positive for *M. morganii*. ^MaE^In one sample, *K. pneumoniae* was detected by LAMP, whereas culture was positive for *E. cloacae* complex.
Mixed cultures	4	2	0	2	^MaE^In two samples, culture was positive for *E. coli* and *K. pneumoniae*, and only *E. coli* was detected by LAMP.
Total	119	105	5	9	

a The discrepancies of these six samples were investigated (see **Table 2**).

b Includes positive results in culture by other microorganisms not included in the panel (two *Streptococcus pneumoniae*, one *Serratia marcescens*, one *Enterobacter cloacae* complex, one *Staphylococcus epidermidis*, and one *Morganella morganii*).

^MiE^Minor errors.

^MaE^Major errors.

### 3.2 Sensitivity determined by the LoD

The estimated LoD for the bacteria included in the detection test was 2.3 × 10^3^ CFU/mL for *S. aureus*, 8.6 × 10^3^ CFU/mL for *E. coli*, 6.7 × 10^3^ CFU/mL for *P. aeruginosa*, 3.6 × 10^3^ CFU/mL for *S. maltophilia*, 1 × 10^5^ CFU/mL for *A. baumannii*, and 9.3 × 10^3^ CFU/mL for *K. pneumoniae*.

### 3.3 Additional molecular method to compare LAMP results

Additionally, to elucidate discrepancies between conventional culture and LAMP assay results, six samples were selected for further study. The BioFire^®^ FilmArray^®^ Pneumonia Panel Plus v2.0 system was employed as a second molecular assay. In all six samples with discrepancies, but one, FilmArray^®^ confirmed the results found by LAMP. In the sample that was not confirmed (sample 104), LAMP was positive for *P. aeruginosa* and *S. maltophilia*, and FilmArray^®^ only confirmed *P. aeruginosa* (**Table 2**).

**Table 2:** Comparison of the results obtained by conventional culture, LAMP, and FilmArray assays in six selected samples.

Sample	Sample type	Culture result	LAMP result	FilmArray^®^ results^a^
20	BAS	*M. morganii*	*S. aureus*	*S. aureus*/*S. agalactiae*
75	EA	*P. aeruginosa*	*P. aeruginosa*/*S. aureus*/*E. coli*	*P. aeruginosa*/*S. aureus*/*E. coli*
82	BAS	*P. aeruginosa*	*P. aeruginosa*/*S. aureus*/*K. pneumoniae*	*Haemophilus* spp./*P. aeruginosa*/*S. aureus*/*S. pneumoniae*/*K. pneumoniae*
109	EA	*P. aeruginosa*	*P. aeruginosa*/*S. maltophilia*	*P. aeruginosa*
111	EA	*P. aeruginosa*	*P. aeruginosa*/*K. pneumoniae*	*P. aeruginosa*/*K. pneumoniae*
117	BAS	*E. cloacae*	*K. pneumoniae*	*E. cloacae*/*K. pneumoniae*

BAS, bronchoaspirate; EA, endotracheal aspirate.

a FilmArray^®^ was used as an alternative molecular method to compare the results obtained by LAMP.

### 3.4 Performance of the LAMP detection panel

For the calculation of panel performance, minor errors were considered as true positives, and mixed cultures were excluded. The sensitivity of the test was 93.33%, whereas the specificity was 92.00%. The PPV was 97.67% and the NPV was 79.31%. The accuracy of panel performance was 93.04% with a kappa value of 0.806 (**Table 3**).

**Table 3:** Summary of the performance metrics and evaluation results for the diagnostic test assessed.

Performance indices	Value
Sensitivity	93.33%
Specificity	92.00%
Positive predictive value	97.67%
Negative predictive value	79.31%
Accuracy	93.04%
Kappa coefficient	0.806

**Figure 2:**
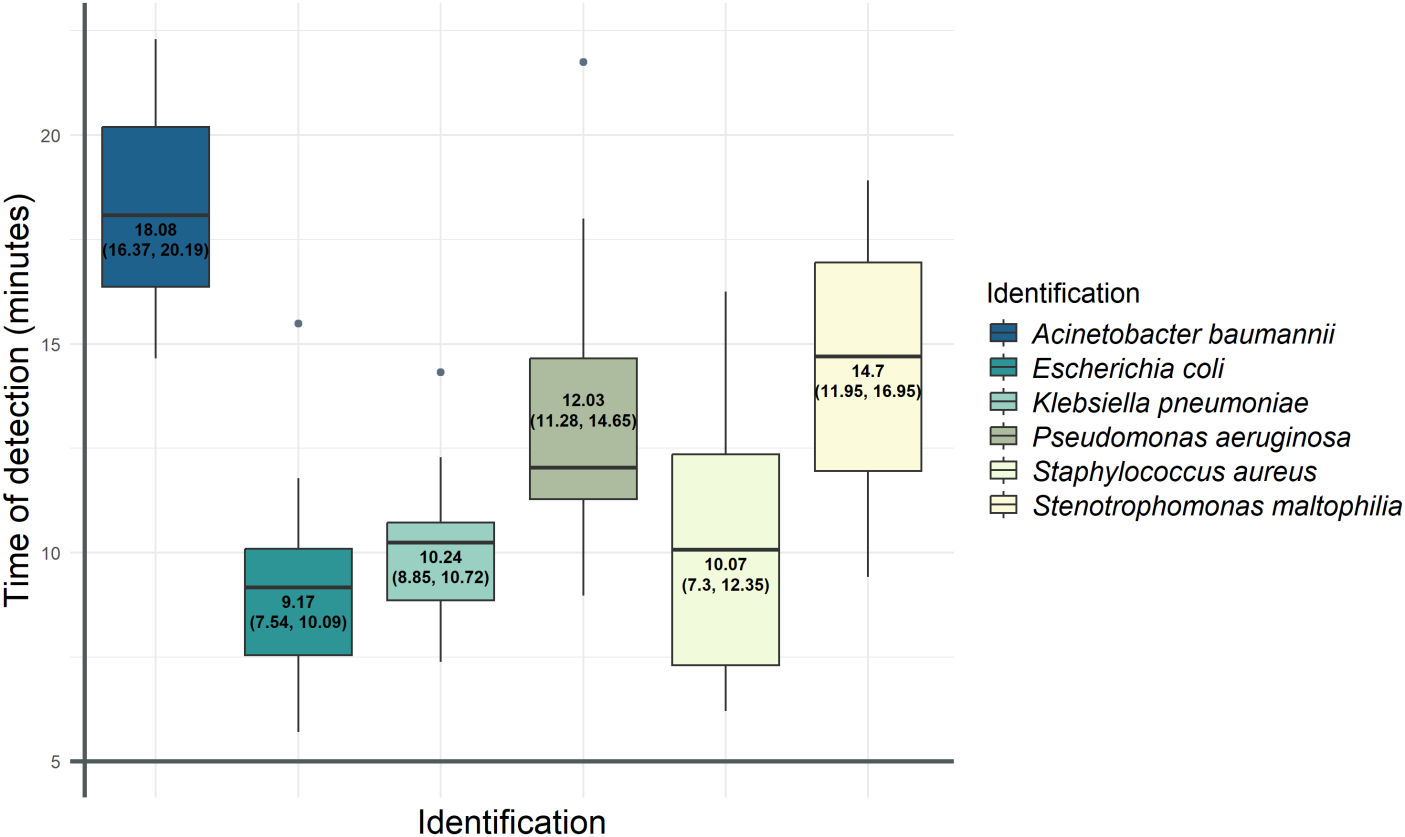
Representation of the media and the first and third quartiles of the positivity time for each bacterium detected by the LAMP technique. For each bacterium detected by LAMP, the mean time of positivity and the IQR were calculated and shown in colored boxes. The values corresponding to the mean, Q1, and Q3 (in brackets) are written inside the boxes. The horizontal line of each of these boxes corresponds to the median detection time; outliers are shown as gray dots.

Among the 119 samples analyzed, the same microorganism was identified in 87 cases by both the LAMP test and the quantitative culture, with a mean detection time of 11.95 min (Q1 = 9.86, Q3 = 14.55). **Figure 2** shows the mean and first and third quartiles for each microorganism included in the test. The time of positivity for *S. maltophilia* presented the highest variability, whereas *K. pneumoniae* showed the most homogeneous time detection.

**Figure 3:**
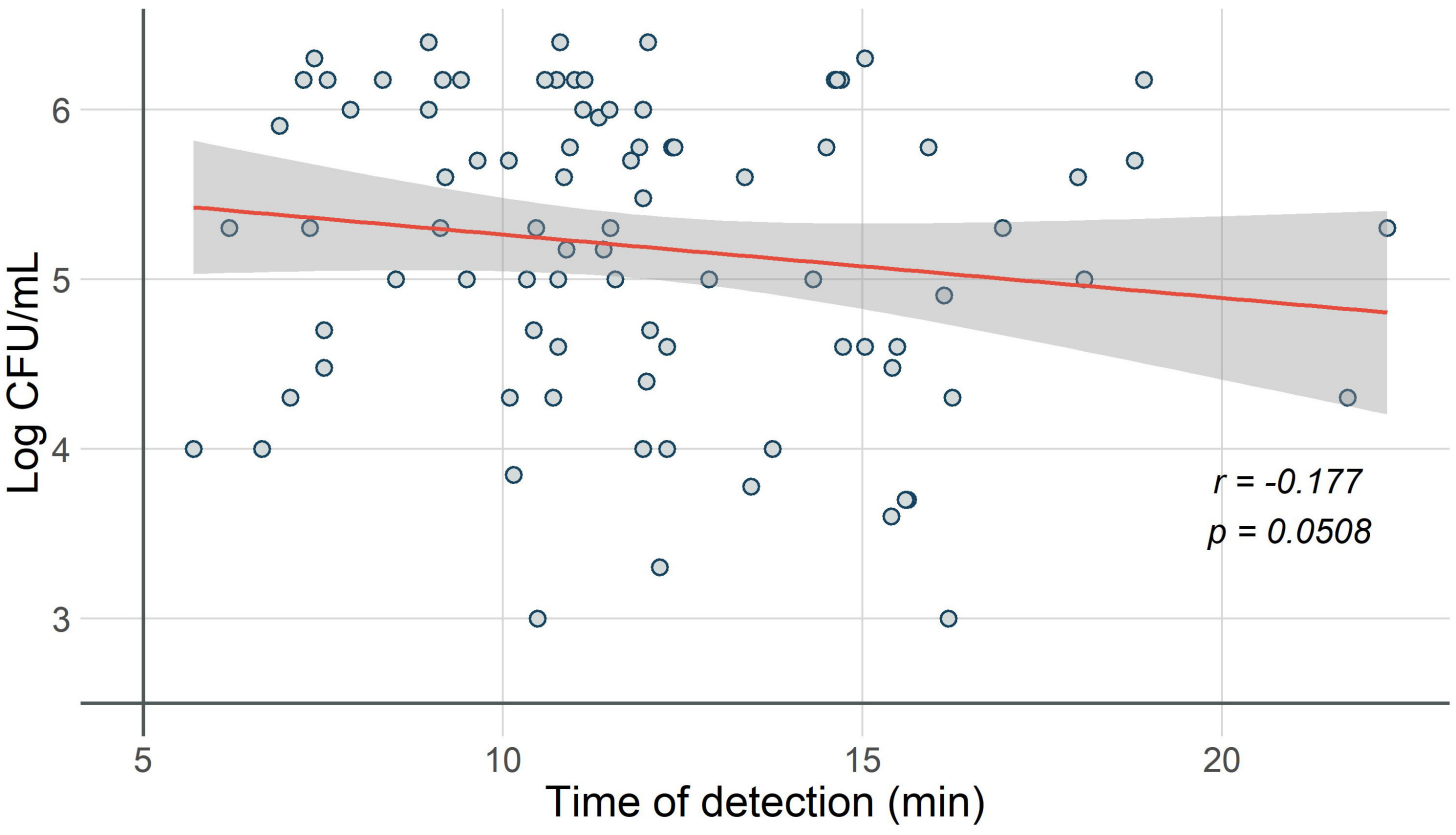
Correlation of time to positivity by LAMP and bacterial load in conventional culture. The correlation between bacterial load (Log CFU/mL) and time to positivity (minutes) is represented in the scatter plot. The trend line shows a weak negative relationship between the variables (*r* = −0.177, *p* = 0.0508), and the area with a 95% confidence interval is indicated (gray area).

The results of the correlation analysis showed a weak negative relationship between bacterial concentrations determined by bacteriological culture and detection time by LAMP (*r* = −0.177, *p* = 0.0508) (**Figure 3**).

### 3.5 Correlating Gram staining with LAMP

Gram staining of respiratory samples was performed as a routine procedure in the microbiology laboratory prior to performing the LAMP assay (**Table 4**). In 86 (72.3%) samples, the Gram staining results were consistent with those obtained from the LAMP assay: in 41 cases, Gram-negative bacilli were observed and detected; in 4 cases, Gram-positive cocci of the staphylococcal type were observed and detected; in 15 cases, mixed microbiota with a predominance of Gram-negative bacilli was observed and one or two Gram-negative bacilli species were detected; in 6 cases, mixed microbiota with a predominance of Gram-positive cocci was found; and in 20 cases, both the Gram stain and LAMP assay results were negative. On the other hand, in 33 samples, the Gram staining results did not correspond with the LAMP assay findings: in 18 Gram stains that appeared negative, the LAMP assay was positive; in 9 samples with observed mixed microbiota, only one microorganism was detected by the LAMP assay; and in 6 samples in which Gram-negative bacilli were observed, the LAMP assay result was negative. However, when we subsequently compared the data from the LAMP assay results with those from conventional culture, in these discordant GRAM/LAMP cases, we found that in 23 out of 33 cases, the microorganism detected by LAMP corresponded to the same isolate identified in the culture.

**Table 4:** Comparison of the results obtained with Gram staining and those from the LAMP assay.

Results of the LAMP assay								
Results of Gram stain	*N*	*P. aeruginosa*	*K. pneumoniae*	*S. aureus*	*E. coli*	*S. maltophilia*	*A. baumannii*	Negative
Gram-negative bacilli^a^	62	29	5^b^	2	7	8	3	8
Gram-positive cocci^c^	10	–	–	9	–	–	–	1
Mixed microbiota	9	1^d^	1	3	2^e^	1	–	1
Negative	38	6	8	3	1	–	–	20
Total	119	36	15	16	10	9	3	30

The results are presented based on the most representative microorganism identified in the assay to facilitate data representation.

a Includes Gram stain of mixed microbiota with a predominance of Gram-negative bacilli.

b One sample mixed with *E. coli*.

c Includes Gram stain of mixed microbiota with a predominance of Gram-positive cocci.

d One sample mixed with *K. pneumoniae*.

e One sample mixed with *S. aureus* and *K. pneumoniae*.

## 4 Discussion

Despite significant efforts to prevent nosocomial infections, HAP remains the second most common nosocomial infection and the leading cause of death from nosocomial infections in critically ill patients (Kalil et al., 2016; Torres et al., 2017). When pneumonia is suspected, it is crucial to initiate appropriate antibiotic therapy as early as possible, as numerous studies have shown that delays in administering the correct therapy result in worse outcomes, increasing healthcare costs and mortality (Rello, 2007; Kuti et al., 2008; Bonine et al., 2019). Therefore, rapid diagnosis by the microbiology laboratory is essential, and several rapid molecular tests are increasingly shortening turnaround times without sacrificing accuracy.

The LAMP panel used in this study included the main bacteria that can cause HAP. We found an overall accuracy of 93.04% between LAMP and culture, and the different performance metrics and evaluation results analyzed showed a strong overall performance with high sensitivity (93.33%) and specificity (92.0%). The PPV (97.67%) suggests that most positive results were related to true infections. The NPV (79.31%) shows that negative results may not always exclude infection. This could be related to the challenges in detecting pathogens at low concentrations or due to the intrinsic characteristics of the sample, which can affect LAMP performance. A larger sample size could help improve the reliability of this metric. The accuracy and the kappa coefficient (0.8) suggest a strong correlation between this test and the gold standard method (culture). These findings are consistent with the data reported in a previous study that used LAMP for the detection of HAP pathogens, which reported an accuracy of 95.2% and a kappa index of 0.89 (Vergara et al., 2020a). This emphasizes the potential of LAMP for use as a point-of-care test.

The target genes used in the kit for species-specific detection were single genes reported to be useful for correct identification in the literature. In *E. coli*, the *pho*A gene is a molecular marker that is well preserved in this species (Elabbasy et al., 2021), while in *A. baumannii*, the gene encoding OXA-51 is an intrinsic and exclusive gene of this species (Turton et al., 2006). For *P. aeruginosa*, the *opr*L gene has been detected in all the strains analyzed in different studies (Chand et al., 2021; Liu et al., 2023). In *S. aureus*, the *fem*A gene codifies an essential protein exclusively presented in this species (Meng et al., 2020), and for *S. maltophilia*, the *stm*Pr gene detected has shown to have a high prevalence in clinical strains (Kang et al., 2012). In the case of *K. pneumoniae* detection, the *pho*E gene has been widely described for its detection in clinical and environmental samples (Sun et al., 2010; Dong et al., 2015). Thus, the use of these target genes has proven to be a reliable tool for the detection of the pathogens analyzed.

*Klebsiella pneumoniae* and *S. maltophilia* were the pathogens in which the most major errors were detected (two each), which could be related to variations in the target genes. In the case of *K. pneumoniae*, it has been reported that genetic variation of the *pho*E gene rarely occurs because of antibiotic exposure (Diancourt et al., 2005; Bialek-Davenet et al., 2017). For *S. maltophilia*, it has been reported that some genes related to virulence, such as *hrp*A, may present variations in their sequence (Youenou et al., 2015) that could affect the detection performance of the primers used. In addition, the presence of LAMP reaction inhibitors in these clinical samples might explain these non-concordant results.

The major errors detected may be related to the amount of pathogen in the samples in which these errors occurred. The sensitivity according to the calculated LoD of the test shows that the sensitivity is adequate and is within the acceptable range reported in the literature, considering that the test analyzed is a rapid detection test (Burd and Kehl, 2011; Xu et al., 2021). Some of the primers used in this kit were previously analyzed, and their LoD was calculated. In the case of *P. aeruginosa* and *K. pneumoniae*, the LoD was 10^2^ CFU/mL, while for *E. coli* and *S. aureus*, the LoD was 10^3^ CFU/mL and 10^4^ CFU/mL, respectively (Vergara et al., 2020a). Comparison of these data with our results indicates similarity. However, in the present study, the LoD calculated for *P. aeruginosa* and *K. pneumoniae* was one-fold higher (10^3^ CFU/mL); in *E. coli*, the LoD range was the same (10^3^ CFU/mL), and the LoD calculated in *S. aureus* was one-fold lower. This could be related to technical and biological factors, such as the device used for the reaction and result analysis. Here, we used a device designed exclusively for use with this kit, and the previously mentioned study used a thermocycler. Furthermore, matrix effects may have influenced detection sensitivity; while the previous study used spiked samples, we used a bacterial suspension made with saline solution. In addition, variations in the LAMP protocol, such as the volumes and concentrations used, may have influenced the different LoDs determined.

It is possible that the pathogen concentrations in the samples in which major errors were detected were low, and this could affect the quantity of DNA extracted, considering that a simple DNA extraction was performed. Moreover, the physical characteristics of the sample could affect the LAMP results. As shown in **Table 2**, all the samples with discrepancies between LAMP and culture results were EA or BAS, which are generally viscous and dense. These characteristics may hinder the effectiveness of the pretreatment done, making it insufficient to fully liquefy the sample, thereby affecting the LAMP results.

It is important to mention that the target panel must be adapted according to the local epidemiology of each hospital ICU and region (Stewart et al., 2021) to cover all the most prevalent pathogens. One limitation of syndromic panels is that they only detect the microorganisms included in the panel, and thus, additional tests or assays may be needed to complement the study. Therefore, it is important to select or design panels that cover the most prevalent microorganisms in our environment. In this sense, it was difficult to collect positive samples for *A. baumannii* for this study, as it is not a very common pathogen in the ICUs of our hospital. Another limitation of this study was the number of samples. For some pathogens, the number of cases isolated was low, and for some sample types such as sputum, the number of samples processed was also low. Having more information about this type of sample is relevant because it may help improve the processing of these non-invasive samples.

An additional interesting study would be to perform a second LAMP assay, after the microorganism causing the infection is known, to determine whether the microorganism carries any known antibiotic resistance mechanisms (extended-spectrum beta-lactamases, carbapenemases, resistance to methicillin, etc.), as done in previous studies (Vergara et al., 2014, 2020b). In the current epidemiological scenario, a significant portion of nosocomial and HAP cases are caused by multidrug-resistant microorganisms (Candel et al., 2023).

The samples used for the validation study in the present study were retrospective; however, the use of prospective samples would help assess the clinical impact of the diagnostic method used. While it may not be cost-effective to use the LAMP test in all samples received in the laboratory, determining which patient populations would most benefit from early diagnosis could be helpful. Gram staining followed by LAMP testing in positive stains could be useful. As observed in this study, in 86 out of 119 cases, the Gram stain result effectively predicted the outcome obtained by the LAMP technique or conventional culture results. These findings support the use of Gram staining as a useful initial screening method for predicting subsequent microbiological outcomes in respiratory samples, which, in addition, would also be more economical. The discrepancies between Gram staining and LAMP findings may stem from the inherent subjectivity and technical sensitivity of Gram staining, especially for respiratory specimens, where studies report up to 42% discordance with culture results (Samuel et al., 2016). In addition, automated systems may reduce variability but still show discrepancies (Froböse et al., 2020). In contrast, LAMP amplifies bacterial DNA directly, allowing the detection of both viable and non-viable organisms with high analytical sensitivity and specificity (Sadeghi et al., 2021). Thus, LAMP-positive/Gram-negative or LAMP-negative/Gram-positive results can reflect true differences in bacterial state or limitations of each method, underscoring the value of combining both approaches for comprehensive diagnosis.

Although the LAMP method does not provide a quantitative measurement of the pathogen, the time LAMP required for achieving a positive result provides a relative estimate of microorganism quantity as observed in this study. However, factors other than bacterial load may affect the detection time, and therefore, it cannot be used as a semiquantitative measure of the pathogen load of a sample. According to our results, most targets were detected within the first 15 min. In other cases, LAMP detected a secondary microorganism not identified by culture: the primary microorganism in the culture samples was isolated in large quantities, possibly masking the lower counts of the secondary microorganism. Other factors, such as the inability of certain microorganisms to grow in culture media or prior antibiotic treatment administered to the patient before sample collection, could affect the LAMP results, similar to what is reported with other molecular techniques (Driscoll et al., 2017; Harris et al., 2017). Notably, these discrepancies could be related to the sensitivity of the LAMP technique, which could be higher compared to culture. This is further supported by the results of FilmArray^®^, which is able to detect a wider range of pathogens than LAMP. This could support the hypothesis that the lack of bacterial growth may be related to factors inhibiting growth, such as previous antibiotic treatment.

Several respiratory syndromic panels based on molecular techniques are available today (Dien Bard and McElvania, 2020; Lade et al., 2022). One advantage of the LAMP technology is its low cost and reduced turnaround time. In previous studies, the cost of the LAMP assay to detect six pathogens was calculated to be 12 euros with a total turnaround time of circa 4 h (Vergara et al., 2020b). Other multiplex panels available are based on PCR, and the sensitivity and specificity of molecular techniques vary widely (Ramanan et al., 2018; Candel et al., 2023). FilmArray^®^ (bioMérieux^®^, Marcy-l’Étoile, France) can simultaneously detect 15 bacteria in a nested-multiplex rt-qPCR, with a turnaround time of 1 h and an estimated cost of 155 euros per sample (Ferrer et al., 2023). In previous studies, the overall sensitivity of FilmArray^®^ for respiratory samples ranged from 75% to 100%, while the specificity varied between 88.9% and 99.5% (Murphy et al., 2020; Enne et al., 2022; Kamel et al., 2022). Unyvero™ HPN (Hospitalized Pneumonia) (Curetis, Holzgerlingen, Germany) enables rapid identification of 29 microorganisms and 19 resistance genes within a turnaround time of 6–8 h, with an overall sensitivity ranging from 55.6% to 100% and a specificity from 14.3% to 99% depending on different factors and the version of the panel (Luyt et al., 2020; Peiffer-Smadja et al., 2020; Darie et al., 2022; Enne et al., 2022). Nevertheless, many of the other panels available primarily focus on identifying viral infections, community-acquired pneumonias, or atypical pneumonias, rather than HAPs (Ramanan et al., 2018; Candel et al., 2023). Moreover, testing frequently relies on nasopharyngeal swabs rather than the more invasive sample types such as those used in this study. This is relevant because invasive samples can generally provide more precise information about the infection since they may contain a higher concentration of the causative pathogen and reduce the presence of the upper airway microbiota, improving diagnostic reliability.

## 5 Conclusions

This study demonstrates that performing the LAMP technique directly on respiratory samples offers a rapid, straightforward, and cost-effective method for identifying the primary bacterial pathogens responsible for HAP. By significantly reducing the time to diagnosis compared to conventional microbiological methods, this approach enables earlier and more targeted antimicrobial therapy. Timely initiation of appropriate treatment is associated with improved patient outcomes, shorter hospital stays, and reduced healthcare costs. Furthermore, integrating such rapid diagnostic tools into clinical workflows can support antimicrobial stewardship efforts by minimizing the empirical use of broad-spectrum antibiotics. These findings underscore the practical value of LAMP as a point-of-care diagnostic tool, with direct implications for frontline healthcare professionals managing hospital-acquired pneumonia.

## Data Availability

The original contributions presented in the study are included in the article/**Supplementary Material**. Further inquiries can be directed to the corresponding author.
