# Supplementary material for: Assessment of a rapid diagnostic test based on loop-mediated isothermal amplification (LAMP) to identify the most frequent pathogens causing hospital-acquired pneumonia
Source: Front Cell Infect Microbiol. 2025 Sep 8;15:1609666. doi: 10.3389/fcimb.2025.1609666 (PMC12450964; doi:10.3389/fcimb.2025.1609666)
Supplement: Supplementary file 1 [file Table1.docx]

**TABLE S1.** Collected samples: type and microbiological findings.

| **Sample** | **Sample type** | **Gram Result** | **Culture Result** |
| --- | --- | --- | --- |
| 1 | EA | Negative | *Klebsiella pneumoniae complex* |
| 2 | EA | GNB | *Pseudomonas aeruginosa* |
| 3 | EA | MM | *Escherichia coli / Klebsiella pneumoniae complex* |
| 4 | BAS | MM | *Klebsiella pneumoniae complex* |
| 5 | EA | GNB | *Pseudomonas aeruginosa* |
| 6 | BAL | GNB | *Pseudomonas aeruginosa* |
| 7 | EA | GNB | *Pseudomonas aeruginosa* |
| 8 | EA | Negative | *Klebsiella pneumoniae complex* |
| 9 | BAL | GNB | *Escherichia coli* |
| 10 | EA | GNB | *Escherichia coli* |
| 11 | EA | MM | *Klebsiella pneumoniae complex* |
| 12 | BAS | GNB | *Stenotrophomonas maltophilia* |
| 13 | EA | Negative | *Pseudomonas aeruginosa* |
| 14 | EA | Negative | *Staphylococcus aureus* |
| 15 | EA | GPC | *Staphylococcus aureus* |
| 16 | EA | GNB | *Klebsiella pneumoniae complex* |
| 17 | EA | GNB | *Pseudomonas aeruginosa / Staphylococcus aureus* |
| 18 | BAS | MM | *Staphylococcus aureus* |
| 19 | BAS | Negative | *Staphylococcus aureus* |
| 20 | BAS | GNB | *Morganella morganii* |
| 21 | EA | Negative | *Morganella morganii / Klebsiella pneumoniae* |
| 22 | BAS | GNB | *Klebsiella pneumoniae / Escherichia coli* |
| 23 | EA | MM | *Stenotrophomonas maltophila* |
| 24 | BAL | GPC | *Streptococcus pneumoniae* |
| 25 | EA | GNB | *Pseudomonas aeruginosa* |
| 26 | EA | Negative | *Klebsiella pneumoniae complex* |
| 27 | BAS | Negative | *Pseudomonas aeruginosa* |
| 28 | EA | GPC | *Staphylococcus aureus* |
| 29 | EA | GNB | *Escherichia coli / Klebsiella aerogenes* |
| 30 | BAS | GNB | *Klebsiella pneumoniae complex* |
| 31 | EA | GNB | *Escherichia coli* |
| 32 | EA | GNB | *Pseudomonas aeruginosa* |
| 33 | BAS | GNB | *Pseudomonas aeruginosa* |
| 34 | BAS | GPC | *Staphylococcus aureus* |
| 35 | EA | GNB | *Pseudomonas aeruginosa* |
| 36 | BAS | GNB | *Serratia marcescens* |
| 37 | EA | GNB | *Pseudomonas aeruginosa* |
| 38 | EA | GNB | *Stenotrophomonas maltophilia* |
| 39 | Sputum | GNB | *Klebsiella pneumoniae / Enterobacter cloacae* |
| 40 | BAS | GPC | *Staphylococcus aureus* |
| 41 | BAS | GNB | *Escherichia coli* |
| 42 | EA | Negative | *Klebsiella pneumoniae complex* |
| 43 | BAS | GNB | *Pseudomonas aeruginosa* |
| 44 | BAS | GNB | *Stenotrophomonas maltophilia* |
| 45 | EA | GPC | *Staphylococcus aureus* |
| 46 | EA | GNB | *Pseudomonas aeruginosa* |
| 47 | BAS | GNB | *Pseudomonas aeruginosa* |
| 48 | BAS | Negative | *Klebsiella pneumoniae* |
| 49 | EA | GPC | *Staphylococcus aureus* |
| 50 | Sputum | GNB | *Pseudomonas aeruginosa* |
| 51 | EA | GNB | *Pseudomonas aeruginosa* |
| 52 | EA | GNB | *Stenotrophomonas maltophilia* |
| 53 | BAS | GNB | *Klebsiella pneumoniae complex* |
| 54 | EA | Negative | *Pseudomonas aeruginosa* |
| 55 | EA | MM | *Escherichia coli / Klebsiella pneumoniae complex* |
| 56 | BAS | Negative | *Pseudomonas aeruginosa* |
| 57 | BAS | Negative | *Pseudomonas aeruginosa* |
| 58 | EA | GNB | *Pseudomonas aeruginosa* |
| 59 | EA | GNB | *Stenotrophomonas maltophilia* |
| 60 | BAS | Negative | *Staphylococcus aureus* |
| 61 | BAS | GNB | *Escherichia coli* |
| 62 | EA | GNB | *Pseudomonas aeruginosa* |
| 63 | EA | GNB | *Stenotrophomonas maltophilia* |
| 64 | BAL | GNB | *Klebsiella pneumoniae complex* |
| 65 | BAL | GNB | *Pseudomonas aeruginosa* |
| 66 | BAL | Negative | *Pseudomonas aeruginosa* |
| 67 | BAL | GPC | *Staphylococcus aureus / Moraxella catharralis* |
| 68 | BAL | GNB | *Pseudomonas aeruginosa* |
| 69 | BAL | GNB | *Staphylococcus aureus / Haemophilus influenzae* |
| 70 | BAL | GNB | *Escherichia coli* |
| 71 | BAL | GNB | *Pseudomonas aeruginosa* |
| 72 | EA | GNB | *Pseudomonas aeruginosa* |
| 73 | EA | GNB | *Pseudomonas aeruginosa* |
| 74 | BAS | GNB | *Stenotrophomonas maltophilia* |
| 75 | EA | Negative | *Escherichia coli* |
| 76 | EA | GNB | *Acinetobacter baumannii* |
| 77 | BAS | GNB | *Acinetobacter baumannii* |
| 78 | EA | GNB | *Acinetobacter baumannii* |
| 79 | BAS | GNB | *Pseudomonas aeruginosa* |
| 80 | BAS | GNB | *Pseudomonas aeruginosa* |
| 81 | BAS | GNB | *Enterobacter cloacae complex* |
| 82 | BAS | GNB | *Pseudomonas aeruginosa* |
| 83 | EA | Negative | *Negative* |
| 84 | EA | Negative | *Negative* |
| 85 | EA | Negative | *Negative* |
| 86 | BAS | Negative | *Negative* |
| 87 | EA | Negative | *Negative* |
| 88 | BAS | Negative | *Negative* |
| 89 | BAS | Negative | *Negative* |
| 90 | EA | Negative | *Negative* |
| 91 | EA | Negative | *Negative* |
| 92 | BAS | Negative | *Negative* |
| 93 | BAS | GNB | *Pseudomonas aeruginosa* |
| 94 | BAS | GNB | *Pseudomonas aeruginosa* |
| 95 | BAS | GNB | *Pseudomonas aeruginosa* |
| 96 | EA | GPC | *Staphylococcus aureus* |
| 97 | EA | GNB | *Pseudomonas aeruginosa* |
| 98 | BAS | GNB | *Haemophilus Influenzae / Streptococcus pneumoniae* |
| 99 | EA | Negative | *Citrobacter koseri / Klebsiella pneumoniae* |
| 100 | BAS | Negative | *Stenotrophomonas maltophilia* |
| 101 | EA | GNB | *Pseudomonas aeruginosa* |
| 102 | BAS | GNB | *Pseudomonas aeruginosa* |
| 103 | BAS | GNB | *Stenotrophomonas maltophilia* |
| 104 | EA | GNB | *Pseudomonas aeruginosa* |
| 105 | EA | MM | *Staphylococcus aureus* |
| 106 | EA | MM | *Pseudomonas aeruginosa* |
| 107 | EA | Negative | *Negative* |
| 108 | EA | Negative | *Negative* |
| 109 | BAL | Negative | *Negative* |
| 110 | BAS | Negative | *Staphylococcus epidermidis* |
| 111 | BAS | Negative | *Negative* |
| 112 | BAS | Negative | *Enterobacter cloacae complex* |
| 113 | BAL | Negative | *Negative* |
| 114 | EA | Negative | *Negative* |
| 115 | EA | GNB | *Stenotrophomonas maltophilia* |
| 116 | EA | GPC | *Staphylococcus aureus / Corynebacterium striatum* |
| 117 | EA | MM | *Staphylococcus aureus* |
| 118 | EA | Negative | *Negative* |
| 119 | BAS | Negative | *Negative* |

BAS: bronchoaspirate, EA: endotracheal aspirate. BAL: Bronchoalveolar lavage. GNB: Gram-negative bacilli. GPC: Gram-positive cocci. MM: Mixed microbiota.
